# Wideband Filtering Slot Antenna Design with Stable Gain Using Characteristic Mode Analysis

**DOI:** 10.3390/s22072780

**Published:** 2022-04-05

**Authors:** Chao Ni, Biyang Wen, Weijun Wu, Ping Ren

**Affiliations:** 1 Radar and Signal Processing Laboratory, Electronic Information School, Wuhan University, Wuhan 430064, China; nichao@whu.edu.cn; 2 Science and Technology on Electromagnetic Compatibility Laboratory, China Ship Development and Design Centre, Wuhan 430060, China; emc1218wu@126.com (W.W.); 13476061860@163.com (P.R.)

**Keywords:** filtering antenna, stable gain, characteristic mode analysis, radiation nulls, magnetic current

## Abstract

A filtering slot antenna with a simple structure combination using characteristic mode analysis (CMA) is proposed. To realize filtering characteristics, characteristic magnetic currents of line and ring slots are analyzed and designed. Then, the folding-line slot and double-ring slot are selected to realize radiation null separately and combined to construct the basic slot antenna. By properly exciting the selected characteristic modes, a wide filtering bandwidth and a stable gain are obtained. To validate the design process, a prototype antenna with a finite ground plane of about 1.1 λ × 1.1 λ is designed and fabricated. Simulated and measured results agree well, which both show a sharping roll rate in the lower and higher frequency and a flat gain realization in the pass band. The filtering bandwidth is 32.7%, the out-of-band suppression level at the higher frequency is over 20 dB, and the gain in the working frequency varies from 3.9 to 5.2 dB.

## 1. Introduction

With the rapid development of wireless communication technology, compact size, high integration, and multiple functions are highly demanded in electronic systems including various RF circuits and components. Therefore, due to the advantages of selectivity, out-of-band suppression, antennas with filtering response and compact size attract lots of attention [1,2,3,4,5,6,7,8,9,10,11,12,13,14,15]. It is common to design filters and antennas separately and cascade them with good impedance matching to achieve filtering characteristics, which inevitably brings insertion loss and introduces extra sizes. Therefore, new co-design methods for filter and antenna integration are proposed. One typical approach for filtering antenna realization is to replace the filter’s last stage resonator with the antenna radiator [6,7,8]. Antennas using this method usually need multiple filter resonators and specific antenna radiator selection, which would introduce extra area and insertion loss. Another approach is to introduce specific element units to realize radiation nulls in the far zone. These units include stacked patch and multiple shorting pins [9], metasurfaces, and parasitic elements [10,11,12]. As this design introduces additional units as well, the whole profile is still high and the design methods are relatively complex. 

Nowadays, due to its direct insight into the antenna radiation principle, CMA becomes popular [16,17,18,19,20,21,22]. It has been found that simple structures without complex combinations can be used to achieve wide bandwidth and other outstanding characteristics [16,17,18,19]. Therefore, with a special design, it is possible to obtain a wideband filtering antenna if the characteristic current of the antenna can cancel each other. However, after our further survey, we find that there are few published reports on filtering antenna realization using CMA. 

In this paper, a simple-structured combined-slot antenna with filtering response is proposed and analyzed using CMA. The antenna employs two simple slot structures including a deformed folding-line slot and a double-ring slot. The deformed folding-line slot is designed from the basic line slot, and the double-ring slot is designed from the single-ring. Using CMA, specific characteristic modes and characteristic magnetic currents are selected to generate radiation nulls. Then, the filtering characteristic is achieved by merging the radiation nulls with a properly designed feeding structure. To demonstrate the design process, a prototype of the antenna is fabricated and measured. Simulated and measured results both indicate that the proposed slot-combined antenna obtains a wide filtering bandwidth and a flat realized gain with sharping roll rate.

This paper is organized as follows. In Section 2, the antenna design stages are given, and two simple slot structures including the folding-line slot and the double-ring slot are analyzed separately using CMA. Then, the filtering antenna with a combination of the above two slots is proposed and its CMs are analyzed to explain the filtering achievement. In Section 3, a prototype antenna is fabricated and measured to verify the total analysis and the design process. Section 4 gives the conclusion of this paper.

## 2. Antenna Design

### 2.1. Antenna Design Stages

**Figure 1:**
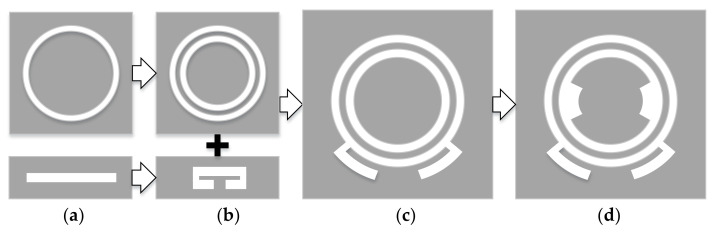
Antenna design stages. (**a**) antenna 1, (**b**) antenna 2, (**c**) antenna 3, (**d**) antenna 4.

It has been proven that combined CMs can be introduced by a probe-fed slot antenna, and the characteristic magnetic currents with proper excitation on the slot antenna can be used to broaden the antenna’s bandwidth with additional stubs [19]. Inspired by the fact that radiation nulls can be generated if electric or magnetic currents on the antenna flow in opposite directions, we consider that slot antennas using CMA can be easily used to bring radiation nulls in specific frequencies. Furthermore, filtering response can be achieved when two radiation nulls are realized separately. Figure 1 shows the filtering antenna design stages. Firstly, two simple structures including a sing-ring slot and line slot are selected. Based on the basic slot structures in Ant.1, Ant.2 can be created to realize radiation nulls using CMA. Then, a double-ring slot and folding-line slot are combined (Ant.3) to achieve filtering characteristics. To improve the impedance matching, Ant.4 (the proposed design) is designed based on Ant.3 by introducing a pair of additional arc-shaped slots inside the inner ring, which are used for impedance matching.

In the following section, a detailed analysis is given using CMA, including a folding-line slot antenna, double-ring slot antenna, and combined-slot antenna. All the simulations performed below were carried out with CADFEKO Suite 7.0. All the cases discussed below are presented on the FR4 substrate, its permittivity *ε_r_* is 4.4 and its height is 0.5 mm. For simulation simplicity, the folding-line slot and double-ring slot are fed by a metal probe, whose both ends are connected with the metal ground. In addition, infinite ground planes were used, and planar Green’s function aperture was adopted to simulate the slot [23]. The radius of the feeding probe for simulation is 0.2 mm. The white part refers to the slot, the gray part refers to the metal ground, and the red line refers to the feeding probe.

### 2.2. Folding-Line Slot 

**Figure 2:**
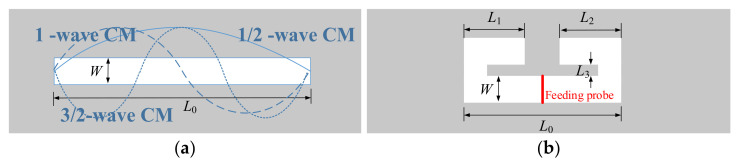
Geometry of the different line-slot antenna. (**a**) line slot antenna, (**b**) folding-line slot antenna.

It is well known that a line slot antenna has a 1/2-wave characteristic mode (CM), 1-wave CM, and 3/2-wave CM at least. Figure 2a illustrates the CMs distribution. It can be observed that the 1/2-wave CM has characteristic magnetic currents flowing in one direction, which is easy to be excited with probe feeding in the middle of the line [19]. For achieving radiation null using this CM, one intuitive idea is to bend two ends of the slot to change the total flowing direction of characteristic magnetic currents. 

Figure 2b shows the ends-folding-line slot antenna. Generally, the length-to-width ratio of the slot should be large enough to be regarded as a thin and linear slot antenna. We chose the line slot length *L*_0_ = 80 mm, the bended part *L*_1_ = *L*_2_ = 32 mm, the length of the joint slot *L*_3_ = 7 mm, and the width *W* = 3 mm. 

**Figure 3:**
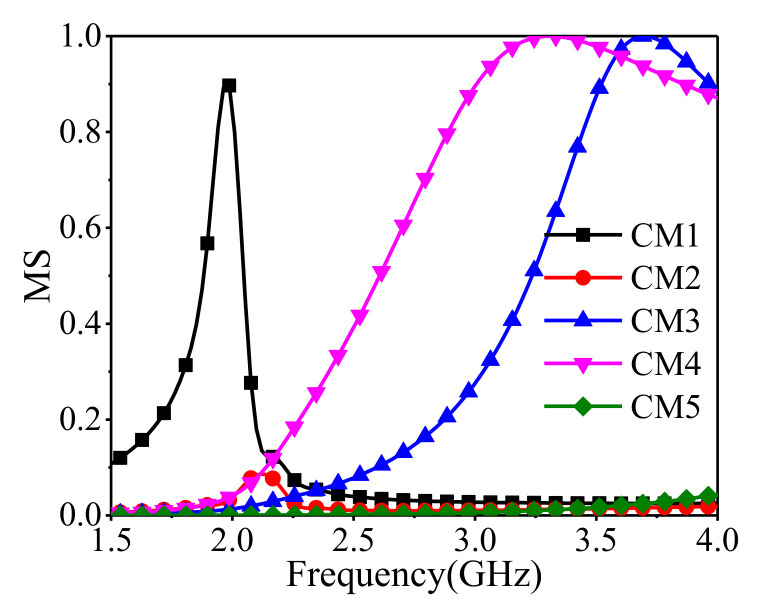
Modal significances of the folding-line slot antenna.

Figure 3 shows the modal significance (MS) curves of the folding-line slot antenna, and three dominant CMs including CM1, CM3, and CM4 can be found. The magnitude of CM2’s MS is too low to radiate.

**Figure 4:**
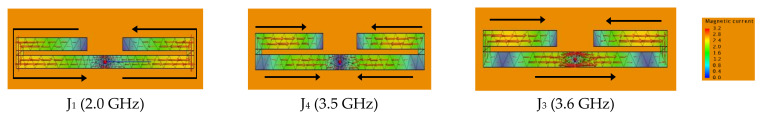
Characteristic magnetic currents of the folding-slot antenna.

Figure 4 illustrates the characteristic magnetic currents of the above three dominant CMs (CM_1_, CM_3,_ and CM_4_) on the folding-line slot antenna. J_n_ represents the modal magnetic current of mode *n* in the slot, and the modal currents are shown at the resonant frequencies. It can be seen that CM_1_, CM_3,_ and CM_4_ are similar to the 1/2-wave CM, 3/2-wave CM, and 2-wave CM on the line-slot antenna. J_1_ and J_4_ flow in opposite directions at two ends of the slot, and the total currents cancel each other, which may be used in radiation null achievement. 

As a narrow line slot structure can be seen as a magnetic dipole, its bandwidth is limited. CM_1_ and CM_4_ cannot be excited to radiate simultaneously within their working band. Due to the simplicity of J_1_’s distribution, CM_1_ can be chosen to achieve radiation null. It is indicated that filtering response on one side of frequency can be realized by adjusting the line slot length and the bent slot length. Together with other filtering structures, the antenna’s filtering characteristics can be realized.

### 2.3. Double-Ring Slot

**Figure 5:**
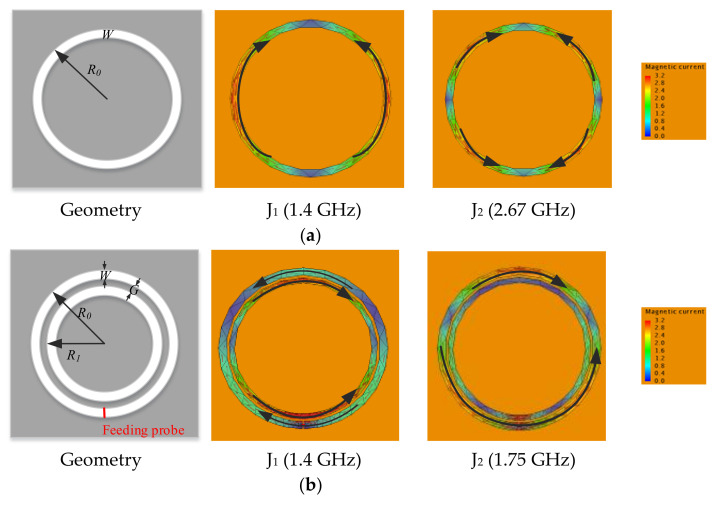
Sing-ring/double-ring slot antenna with *R*_0_ = 32.3 mm, *W* = 3 mm, *R*_1_ = 28.3 mm, *G* = 1 mm, and corresponding characteristic magnetic current distribution of two typical CMs.

Figure 5a shows the geometry of a typical single-ring slot antenna and its main CMs’ magnetic currents at resonant frequencies. It can be observed that a single-ring slot antenna is hard to generate radiation nulls in the lower frequency, as the corresponding characteristic magnetic currents in the lower frequency flow towards one direction. In addition, it is hard to excite high-order CM as the characteristic magnetic currents are complex. For achieving filtering characteristics, one intuitive idea is to insert one ring-slot into another to form an opposite current flow.

Figure 5b shows the double-ring slot antenna. It consists of two ring slots with a fixed gap *G*. The radii of the outer and inner ring slots are *R*_0_ and *R*_1_ respectively, and the width of the slot is *W*. As the inner ring slot is not easily fed, the feeding probe is placed across the outer ring. It can be observed that the characteristic magnetic current distribution of the double-ring slot changes a lot compared with the single-ring slot. The total eigencurrents of CM_2_ flow in one direction, and it is not in the aimed mode. 

CM_1_’s characteristic magnetic currents in the inner and outer ring slots flow in opposite directions. Besides, J_1_ is mainly distributed in the inner ring. With the probe feeding across the outer ring, magnetic currents in the outer ring can be improved. Together with the excited CM_1_, magnetic currents in the inner and outer ring slot can be canceled, and radiation null can be realized. Therefore, the double-ring slot can be used with other structures to form a better filtering antenna due to the simplicity of the geometry.

### 2.4. Working Principles of the Proposed Antenna

**Figure 6:**
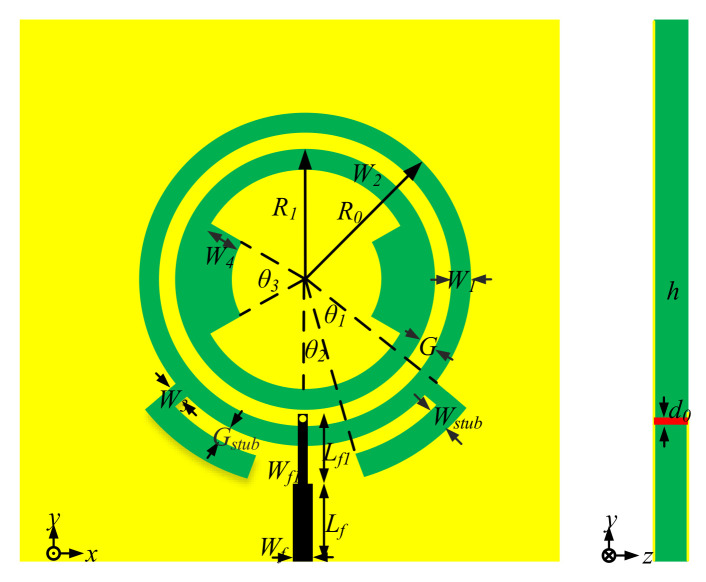
Proposed filtering antenna. *R*_0_ = 21.8 mm, *R*_1_ = 17.5 mm, *W*_1_ = 3 mm, *W*_2_ = 2 mm, *W*_stub_ = 1.8 mm, *W*_3_ = 6 mm, *G*_stub_ = 1 mm, *G* = 1 mm, *W*_4_ = 3 mm, *θ*_1_ = 52°, *θ*_2_ = 10°, *θ*_3_ = 60°, *h* = 0.5 mm, *L_f_* = 24 mm, *L_f1_* = 8.2 mm, *W_f_* = 0.92 mm, *W_f1_* = 0.5 mm, *h* = 0.5 mm.

To achieve a good filtering response, the above structures are combined together. The geometry of the proposed antenna is given in Figure 6. The antenna is printed on an FR4 substrate, its permittivity *ε*_r_ is 4.4, and its height *h* is 0.5 mm. It has a dimension of 100 mm × 100 mm. The yellow, green, and black parts refer to the metal ground, FR4 substrate, and the stepped metal microstrip line used for 50 Ω excitation lying on the back side of the substrate. The end of the feeding-line is connected with the ground plane via a metal shorting pin, which is shown in red color. The proposed feeding structure with the shorting pin is similar to the above probe-fed structure [18], which is easier to be fabricated and ensures the whole slot structure's excitation.

**Figure 7:**
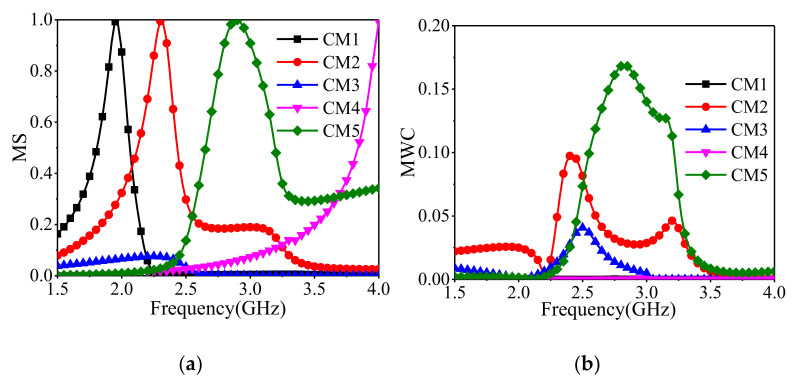
Modal significances and modal weighting coefficients for the first five modes of the proposed antenna. (**a**) modal significances, (**b**) modal weighting coefficients.

**Figure 8:**
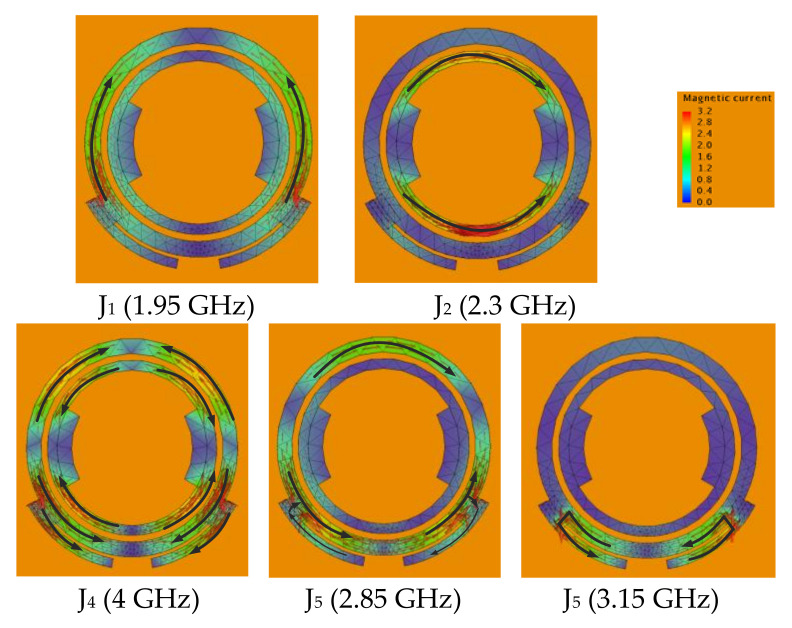
Magnetic eigencurrents of CMs.

Figure 7 and Figure 8 show the modal significances of the first five CMs and the corresponding magnetic eigencurrents. CM_3_’s MS is too low to be radiated. CM_1_ and CM_4_ are distributed at both ends of the frequency band, the resonant frequency difference is beyond 2 GHz, and they are hard to be excited at the same time. Figure 7 also depicts the modal weight coefficient (MWC) curves of the first five CMs. It can be seen that CM_2_ and CM_5_ are mainly excited within the aimed frequency range. Although other CMs such as CM_3_ are excited, their magnitude of MS is too low to be radiated. Therefore, only CM_2_ and CM_5_ should be considered.

It can be seen that magnetic eigencurrents of CM_2_ and CM_5_ are mainly distributed in the inner-ring and outer-ring slots separately. CM_2_ is similar to the corresponding CM_1_ of the double-ring slot. CM_5_ is a new CM introduced after slots combination compared with the double-ring slot and is similar to the superposition of corresponding CMs of the above slots. The resonant frequency spacing between CM_2_ and CM_5_ is nearly 500 MHz. With the proposed feeding structure, CM_2_ and CM_5_ are mainly excited, and wide bandwidth can be achieved. Meanwhile, as magnetic currents in the outer ring and slot stubs are excited and enhanced, a radiation null in the lower frequency can be achieved for CM_2_, just like CM_1_’s behavior in the double-ring slot. As shown in Figure 8, for CM_5_ in the upper frequency, magnetic current distribution in the outer-ring and slot stubs changes, and the eigencurrents are mainly distributed in the slot stubs. Similar to CM_1_’s behavior in the folding-line slot, it brings another radiation null. Therefore, we can adjust the frequency band utilizing CM_2_ and CM_5_ by changing the proper elements of the proposed antenna.

Besides, characteristic magnetic currents of excited CM_2_ and CM_5_ flow in one direction, which ensures the antenna has a good linear polarization. 

**Figure 9:**
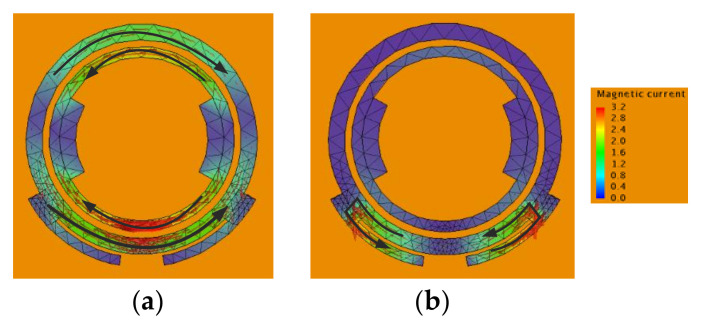
Simulated magnetic current distribution of the proposed antenna at different frequencies. (**a**) 2.08 GHz (**b**) 3.23 GHz.

**Figure 10:**
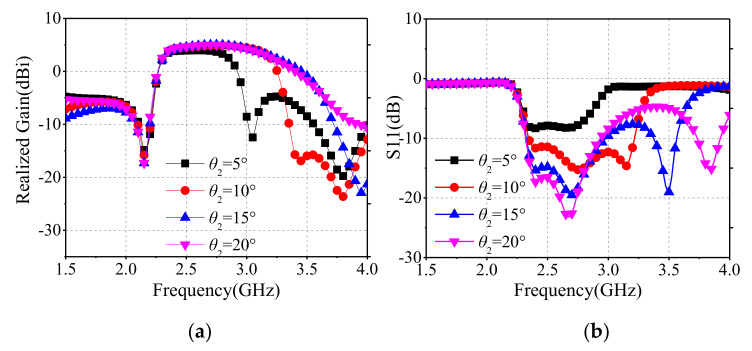
Simulated results for different parameter *θ*_2_, (**a**) realized gain, (**b**) S11.

Figure 9 shows the magnetic current distribution at the first depression frequency in the lower and upper bands. It can be easily seen that the filtering response in the lower and upper bands is predominantly realized by the excited magnetic currents in the double-ring and folding-line slot separately. This agrees with the former analysis. Figure 10 gives the simulated S11 and realized gain. It can be seen that the realized gain has a sharp rolling rate at the upper and lower frequencies, which shows a high out-of-band suppression characteristic and a good filtering response within the 10 dB impedance bandwidth.

To further illustrate the proposed antenna’s mechanism, we choose *θ*_2_ and *W*_4_ for parameter sweep analysis. Figure 10 shows that the high out-of-band suppression at the higher frequency varies with *θ*_2_. This is because that *θ*_2_ is associated with the total length of the folding-line slot. As *θ*_2_ increases, the total length of the folding-line slot decreases, and the corresponding resonant frequency increases.

**Figure 11:**
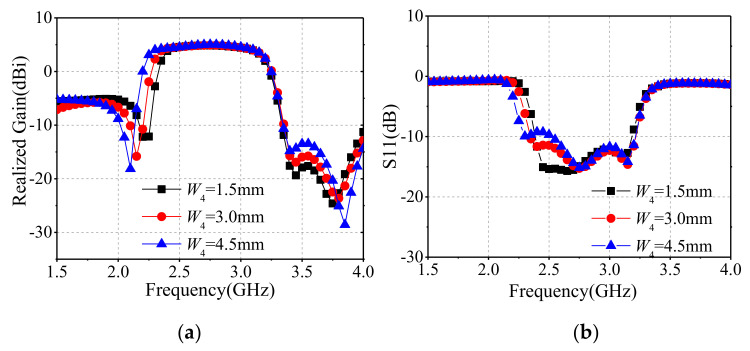
Simulated results for different parameter *W*_4_, (**a**) realized gain, (**b**) S11.

Figure 11 depicts the filtering bandwidth that varies with *W*_4_ around lower frequencies. *W*_4_ is the width of the inner arc-shaped slot stub. As characteristic magnetic currents of CM_2_ and CM_5_ around the inner arc-shaped slots are rare, *W*_4_ can be chosen to adjust the impedance bandwidth, and the corresponding filtering frequency varies.

## 3. Results and Discussion

According to the analysis results mentioned above, the designed antenna was prototyped and measured. 

**Figure 12:**
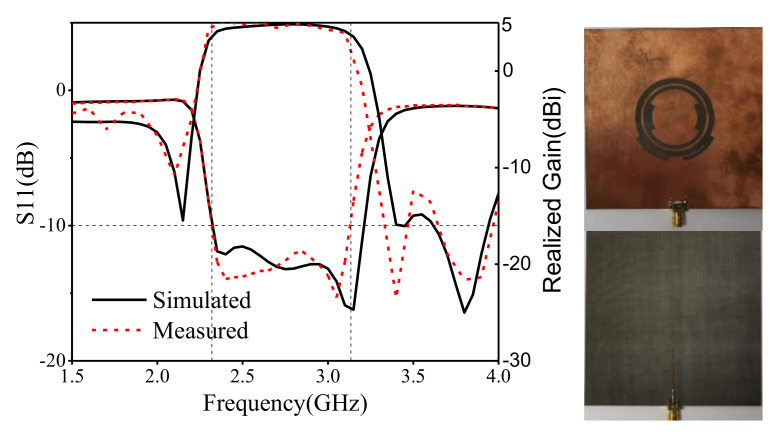
Comparisons of the simulated/measured S11 and realized gain, prototype of the antenna.

**Figure 13:**
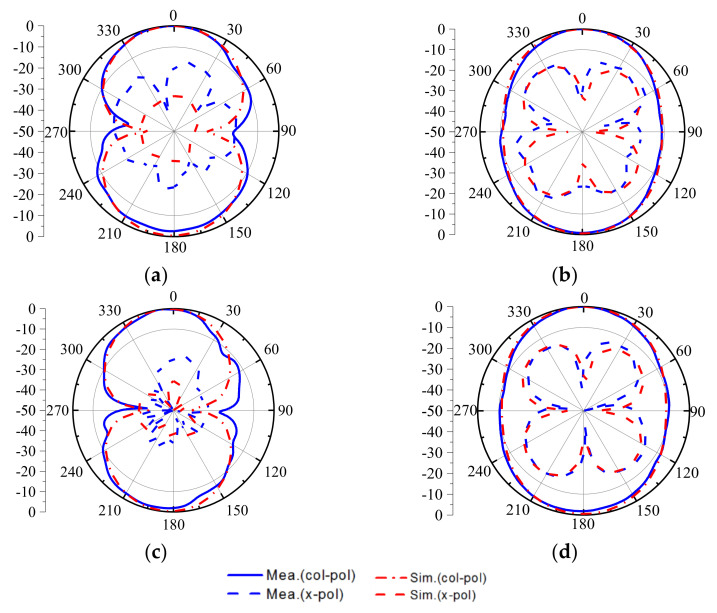
Simulated/measured radiation patterns of the proposed antenna, (**a**) xoz plane@2.5 GHz (**b**) yoz plane@2.5 GHz (**c**) xoz plane@3 GHz (**d**) yoz plane@3 GHz.

Figure 12 gives the comparison between the simulated and measured results of S11 and realized gain. It can be seen that the measured bandwidth ranges from 2.32 GHz to 3.13 GHz, and the simulated and measured results agree well. The gain ranges from 3.9 dB to 5.2 dB, and the average suppression around the upper frequencies is above 20 dB, which demonstrates the filtering characteristic. The measured S11 and gain shift a little left around 3.2 GHz. The difference is mainly caused by manufacturing errors and welding deviation. Figure 13 gives the radiation patterns of the measured and simulated results in different frequencies. It shows that the proposed antenna has a good broadside radiation characteristic and a stable radiation pattern across the whole working bandwidth. The cross-polarization suppression levels at different planes are mostly lower than 20 dB, but a little higher at the upper frequencies, which is caused by the imperfect measurement environment and the connected SMA. 

Comprehensive comparisons with reported filtering antennas of different types are given in Table 1. In [1,10], complex structures including stacked patches with specific shorting pins and metasurface design are utilized. In [2], a conventional patch antenna with designed etched slots is used to achieve filtering response, but the bandwidth is limited. Due to its 2-order filter design, the filtering antenna in [6] shows a good stable gain, but a limited bandwidth and complex structure. It can be seen that the proposed filtering antenna using CMA has advantages of low profile, simple structure, wide bandwidth, and stable gain.

## 4. Conclusions

In this paper, a combined-slot antenna with filtering characteristics and stable gain using CMA is proposed. With CMA, double-ring slot and folding-line slot are analyzed to achieve radiation nulls at different frequencies. By combining the above slot structures, two radiation nulls can be realized. Further, two CMs with proper excitation are selected to obtain wide bandwidth. A fabricated prototype achieves a wide bandwidth of 32.7%, and a stable gain of 3.9~5.2 dB, which demonstrates the design process. Compared with other filtering design antennas, the proposed one exhibits the advantage of wide bandwidth, low profile, and simple structure, which shows the potential to be used in high-integrated microwave systems.

## Figures and Tables

**Table 1:** Comparisons of filtering antennas.

Reference	Configuration	Size (λ_0_^3^)	BW	Gain (dBi)
[1]	Stacked slot patch with shoring pins	1.13 ∗ 0.42 ∗ 0.06	23.5%	4.7~7.5
[2]	Single patch with etched slots	0.7 ∗ 0.59 ∗ 0.03	7.1%	4.6~6.6
[6]	Single slot patch with n-order filter	0.25 ∗ 0.3 ∗ 0.02	18.9%	0.7~2.3
[10]	Metasurface with designed slots	0.77 ∗ 0.77 ∗ 0.04	17.6%	7~9
Prop.	Single combined slot patch	1.1 ∗ 1.1 ∗ 0.01	30.6%	3.9~5.2

## Data Availability

The data supporting this research article are available upon request to the author.
